# Compliance Audit of Processed Complementary Foods in Urban Ghana

**DOI:** 10.3389/fpubh.2015.00243

**Published:** 2015-10-27

**Authors:** Richmond Nii Okai Aryeetey, Marcella Tay

**Affiliations:** ^1^Population, Family, and Reproductive Health Department, University of Ghana School of Public Health, Accra, Ghana

**Keywords:** complementary foods, labeling, marketing, messages, Ghana, compliance

## Abstract

**Background and objectives:**

Although processed complementary foods (PCFs) can contribute to meeting dietary needs of infants and young children, it has been associated with unethical marketing practices, which undermine practice of exclusive breastfeeding for 6 months. The current study assessed PCF labeling compliance to the International Code of Marketing of Breast Milk Substitutes (CMBMS) and the National Breastfeeding Promotion Regulation (NBPR) in Ghana.

**Methods:**

A variety of PCF were purchased from child welfare clinics, fuel station shops, supermarkets, “mother/baby” care shops, and pharmacies in the La and Osu Klottey sub-metropolitan areas in Accra. The labels were evaluated against the best practice indicators proposed by the Maternal, Infant, and Young Child Nutrition Working Group based on the international CMBMS, and also indicators based on the NBPR. An overall compliance estimate was determined based on intensity of compliance to the indicators.

**Results:**

The PCF purchased included cereal-based products, fruit juices, fruit and vegetable purees, milk-based products, and combination meals; 75% of PCF were imported. One hundred of the 108 products identified were labeled in English and thus included in analysis. None of the products complied with all labeling requirements of CMBMS or NBPR; 84 and 17% of product labels complied with at least 50% of NBPR and 50% of CMBMS indicators, respectively. Only 5% of labels had content indicating importance of exclusive breastfeeding for 6 months. Additionally, only 5% of labels warned against the hazard of introducing PCF earlier than 6 months as required by the NBPR.

**Conclusion:**

Labeling of most PCF sold by selected retailers in Accra did not comply with NBPR and CMBMS labeling requirements. Enforcement of local law on labeling of PCF is urgently needed.

## Introduction

The World Health Organization (WHO) recommends exclusive breastfeeding of infants for the first 6 months of life (1, 2). This is because breast milk provides adequate amounts of the essential nutrients needed by infants up to the first 6 months of life (3–6). Thereafter, introduction of complementary foods is recommended, since breast milk alone is no longer sufficient to meet the requirements for optimal growth and development (7, 8). Early introduction of complementary food (before the child turns 6 months old) is, however, considered inappropriate; there is evidence that early introduction of complementary foods interferes with optimal breastfeeding (9, 10).

Furthermore, in developing country settings where risk of infectious morbidity is often high (11), poor quality complementary foods (having low-nutrient density), together with inappropriate complementary food administration practices, contributes significantly to early undernutrition in infants and young children (12, 13). Young children who miss the opportunity to be fed with appropriate complementary foods of adequate nutrient quality are likely to suffer irreversible damage to their physical and mental development (12, 13).

There are situations where local and home-prepared foods alone, is insufficient to meet the nutritional needs for complementary feeding (1). In such situations, processed complementary foods (PCFs) of adequate nutrient quality are recommended (1). It is also recommended that these foods should meet the applicable standards of Codex Alimentarius Commission, including the Codex Code of Hygienic Practice for Foods for Infants and Children (14, 15).

Furthermore, the global nutrition community has not forgotten the recent history of inappropriate and aggressive marketing of infant formula, leading to inappropriate child feeding practices, increased malnutrition, and the associated elevation in child mortality, especially in the developing countries (16). It was in response to such inappropriate involvement of business in child nutrition that in 1981, the International code of marketing of breast milk substitutes (CMBMS) was instituted to protect, promote, and support breastfeeding among infants and young children. The CMBMS provides guidance on regulating the inappropriate marketing of breast milk substitutes, teats, and bottles used for the feeding of young children (17).

It is unfortunate that despite the existence of the CMBMS and its wide acceptance by many countries, such inappropriate marketing practices continues be reported, even today (18, 19). Inappropriate promotion and labeling practices in the marketing of PCFs also have the potential to undermine optimal breastfeeding practices and does result in infant malnutrition, morbidity, and mortality (20). Infant formula manufacturing companies have been shown to provide misleading information on infant formula labels (21).

In the case of breast milk substitute marketing, the CMBMS is the key guidance for monitoring appropriate promotion and marketing. However, a similar guidance tool is not available in the context of complementary food marketing. In the interim, principles of the CMBMS remain a useful guidance. However, gray areas in monitoring remain, regarding foods targeted at young children who are about to start complementary foods but are still breastfeeding. There is some evidence suggesting that baby food manufacturing companies are taking advantage of the emphasis the CMBMS places on infant formula to market PCFs, inappropriately (22, 23). In the absence of clear guidelines building on and strengthening the CMBMS, and which focuses on the marketing of PCFs, caregivers of young children who are yet to be introduced to complementary feeding, are at risk of being misled to commence complementary feeding earlier than recommended.

In order to bridge this gap in the regulation of marketing of complementary foods, the Maternal, Infant, and Young Child Nutrition (MIYCN) Working Group of the “10-Year Strategy to Reduce Vitamin and Mineral Deficiencies,” has proposed a framework to guide the marketing of PCF (24). The proposed framework is based on the principles of the international CMBMS and among other things, and the proposed “indicators” that may be used to guide appropriate marketing and labeling of complementary foods, which are targeted at young children. Although the proposed framework is the first of its kind, globally, it is not yet adopted by consensus.

An additional existing mechanism to determine compliance to the best practices in the marketing of complementary foods is the adaptation of the CMBMS into local laws in various countries, as the case is in Ghana. In the year 2000, the Government of Ghana passed the National Breastfeeding Promotion Regulation (NBPR) (25). The NBPR is based on the principles of the CMBMS and specifically identifies breast milk substitutes generally as “designated products.” The regulation has established provisions covering the promotion, distribution, display of related printed material in health facilities, labeling, and companies’ relationships with health personnel in respect of designated products. However, like the international CMBMS, NBPR does not have specific provisions for regulating the marketing of processed commercial complementary foods targeted for feeding children at 6 months old or beyond. As a result, a gray area remains that is subjected to exploitation by companies, which produce or market complementary foods. Between November 2004 and June 2005, only 25% of infant formula labeling in Ghana complied with relevant national guidelines (26). In exploiting this gray regulatory area, caregivers may be led to believe that children could commence complementary feeding, even though by doing so, they may be failing to meet the globally accepted recommendations for feeding infants and young children.

The current study was, therefore, designed to describe compliance of PCF marketed and distributed in urban Ghana based on (1) indicators proposed by the `MIYCN working group and also (2) indicators relevant to this assessment that already exists in the NBPR. The study findings identified potential gaps in the labeling of PCFs marketed in Ghana.

## Materials and Methods

The study was carried out between May and June 2012. Data were collected from labels of PCF for young children, marketed in Ghana. Product samples were purchased from selected vendors in the La and Osu Klottey Sub-Metropolitan administrative areas in Accra, the capital city of Ghana. The selected vendors included government child welfare clinics (CWCs), supermarket stores, pharmacies/chemical stores, “mother care” shops (selling mainly items targeting infant and young child care, including formula and other foods designed for young children) (19), and also fuel vending stations in the study areas in Accra. Two CWCs were selected purposively, one from each sub-metropolitan administrative area. Other vendors were selected based on the size and popularity of the store. The selection of PCF vendors was planned to ensure comprehensive coverage of complementary foods available to buyers. It was assumed that larger and popular stores were more likely to be patronized by retailers; and therefore these were prioritized for vendor selection. All uniquely labeled PCF were considered eligible for inclusion in the assessment. At selected vendors, a sample of all uniquely labeled PCF, displayed in the baby/child products section of the shop/vending site, or having labeling information targeted at children, was purchased for assessment. Uniquely labeled PCF included those whose label displayed information showing different flavors, and formulation, irrespective of brand name or manufacturer. Different size of packaging was not a differentiating factor.

Using a previously utilized approach (27), the labels on the complementary food were evaluated based on the indicators proposed in the draft guide for marketing complementary foods as well as similarly constructed indicators identified from the NBPR (Table 1) (24). Briefly, each label was compared with each of the compliance indicators of both the MIYCN working group (11 indicators) and the NBPR (6 indicators). An extraction tool was used to obtain information from the PCF labels. A checklist was used to assess the compliance of the complementary food labels to the labeling indicators. Table 1 shows the criteria for determining compliance for each indicator. Ethical clearance for the study was obtained from the Ghana Health Service Ethical Review Board.

**Table 1:** **Checklist showing compliance indicators and criteria determining compliance**.

Compliance indicators	Answers	Criteria for answer
Product labeled in appropriate language (English)	Yes	Information on label is written in English
	No	Information on label is not in English
Label text is clear and conspicuous	Yes	Information on label is at least 5 mm in height
	No	Information on label is <5 mm in height
Label information is visible prior to purchase	Yes	Label information is not in an under-lid leaflet or a detachable leaflet prior to purchase
	No	Label information is in an under-lid or detachable leaflet prior to purchase
Label shows pictures of children 6 months old	Yes	Picture shows baby performing a milestone achieved after the age of 6 months for example standing alone
	No	Picture shows baby performing a milestone usually associated with infants 0–6 months such as sitting without support
	Not applicable cannot be determined	No picture of a baby is shown on the product label
		Picture shown on label is ambiguous on the intended age of the baby. For example, child sitting in high chair holding a spoon
Label specifies an age that does not precede 6 months	Yes	Recommended age of introduction is at least 6 months
	No	Recommended age of introduction is <6 months
	Not Applicable	The label does not specify an age of introduction
Label emphasizes the importance of exclusive breastfeeding for first 6 months	Yes	The message should include the following concepts: Exclusive breastfeeding and first 6 months
	No	The label does not make a statement with the concepts mentioned above
Label indicates that continued breastfeeding should be from 6 months to 2 years and beyond	Yes	The message should include the following concepts: continued breastfeeding up to 2 years
	No	The label does not make a statement with the concepts mentioned above
Label indicates that product should be fed with a spoon	Yes	Label information should contain one of the following: (i) show a picture of a spoon or actually state that (ii) the product should be fed with a spoon
	No	Label does not contain any of the above
Label should not give instructions on how to feed product with a feeding bottle	Yes	Label should not show any of the following: a picture of a bottle or actually state that the product should be fed in a bottle
	No	Label shows a picture of a bottle or actually states that the product should be fed in a bottle
Label proposes a daily ration	Yes	Label should state one of the following: (i) the amount and (ii) the number of times the product should be fed to the baby
	No	Label does not provide information on the amount and number of times the product should be fed
Label stipulates warnings	Yes	Label information should contain one of the following: (i) hazards of inappropriate preparation, (ii) hazards of inappropriate storage, and (iii) hazards of inappropriate use
	No	Label does not contain information on any of the above information
Label shows pictures required only for illustration of the method of preparation	Yes	Label information should contain one of the following: (i) show pictures providing information on preparation only and (ii) not show pictures of babies performing any milestone
	No	Label shows pictures for illustrating method of preparation as well as developmental milestones of babies

### Analysis

Uniquely labeled processed food products marketed for the purpose of feeding young children were purchased from the listed vendors. The products were considered unique if the label displayed information showing different flavors, and formulation, but not size of packaging, irrespective of brand name. A checklist based on the criteria for assessing processed food marketing based on the MIYCN proposed indicators was used to extract the needed information on each product. Criteria were set for each indicator as shown in Table 1. Similar criteria were used for extracting compliance data for the NBPR (25). Percentages were used to summarize the compliance of each product label per indicator.

## Results

The study identified 108 unique complementary food products from 30 different manufacturers (17 local and 13 foreign manufacturer brands). The product categories identified are presented in Table 2. The products included cereal-based products, milk-based products, and pureed foods. The cereal-based products identified had two subgroups consisting of ready-to-use foods and those that require heat treatment. About half of the products were cereal-based, whereas milk-based products made up about 5% of the products identified. The products were packaged in jars, bottles, plastic wraps, aluminum tins, and paper-laminated boxes.

**Table 2:** **Categories of processed complementary food products identified in Accra (*N* = 108)**.

Food product characteristics	Number	%
**Product category**		
Cereal-based products	53	49.1
Fruit juices	7	6.5
Fruit and vegetable purees	29	26.9
Milk-based products	5	4.6
Pureed foods^a^	14	12.9
**Preparation method**		
Ready to use	82	75.9
Requires heat treatment	26	
**Product origin**		
Locally manufactured	27	25.0
Imported	81	75.0
**Production information**		
Number of manufacturers	29	26.8
Products including manufacturing date	98	90.7
Products including expiry date	98	90.7
Total	108	100

*^a^Pureed foods were either fruits or vegetables; vegetable purees may contain a source of animal protein*.

Table 3 shows the compliance of PCFs to the indicators of the two sets of guidance documents. Some indicators were common to both the MIYCN working paper and the NBPR. None of the products complied with all labeling requirements of the MIYCN or NBPR indicators; 84 and 17% of product labels complied with at least 50% of MIYCN and 50% of NBPR indicators, respectively. Both category of indicators required that the product labels should be in English, be clearly readable, and provide guidance for users on a daily minimum serving of the product. In addition, the indicators required inclusion of instructions for the safe preparation, use, and storage of the product. Of the 108 products purchased, 93% were in English language. The eight products, which were not in English, were not included in the label compliance assessment.

**Table 3:** **Compliance of food labels to proposed indicators of appropriate labeling of processed commercial complementary foods in Accra, Ghana (*N* = 108 products)**.

Indicators of appropriate labeling of complementary food product	Number of compliant products	Product compliance (%)	Number of products evaluated
**Indicators based on the miycn working paper**			
Product label in local language (English)^a^	100	93.0	108^b^
Label text is clear and conspicuous (clearly readable)^a^	6	6.0	100
Label information is visible before purchase	98	98.0	100
Label specifies an age of introduction that is 6 months and above	77	96.0	80^d^
Label shows babies appearing to be older than 6 months^e^	10	14.0	72^c^
Label emphasizes importance of exclusive breastfeeding for first 6 months^e^	5	5.0	100
Label indicates that continued breastfeeding should be for 2 years or beyond^e^	37	37.0	100
Label proposes daily ration/serving^a^^,^^e^	29	29	100
Label includes instructions regarding safe preparation, use, and storage of product^a^	91	91	100
Label does not recommend feeding product in a bottle	28	98	100
Label recommends feeding the product with a spoon	28	28	100
**Indicators based on the breastfeeding promotion regulation**			
Label has a message that breast milk is the best food for infant and prevents diarrhea and other illnesses	1	1	100
Label includes a warning against the health hazards of improper preparation and use of the product^a^	7	7	100
Label does not show any photograph or other graphical representation other than for illustration the method for preparation	27	27	100
Label has the name and address of the manufacturer/distributor	98	98	100
Label has the composition and contents of the product	98	98	100
Label has a batch number	92	92	100

*^a^Indicators common to both the working paper and the regulation*.

*^b^Total number of products purchased*.

*^c^The number of product labels that had pictures*.

*^d^The number of product labels that suggested an age of introduction*.

*^e^Indicators recommended by the working paper for optimal infant feeding and are in full compliance with the Code of Marketing of Breast Milk Substitutes, but are not explicitly required*.

### Messages on Breastfeeding

Almost all the labels (96%) met the MIYCN indicator requirement to specify the age for introducing the product as beyond age 6 months (Table 3). However, only 5% of labels had a message indicating the importance of breastfeeding during the first 6 months of life. Furthermore, only one label met the NBPR indicator requirement to indicate that breast milk is best food for preventing diarrhea and other child illness. Also, only about one-third (37%) of labels provided messages on continued breastfeeding for the first 2 years of the child’s life.

### Images on the Label

Of the 100 evaluated labels, 27 did not include any images of children. Using the MIYCN indicator criteria, only 14% of the 72 child images displayed were judged to be clearly older than 6 months old.

### Instructions on Preparation and Feeding

More than 90% of labels had instructions on preparing the food product. However, the requirement by NBPR to have warning messages regarding improper preparation of the food product was only met by 7% of labels. Furthermore, only 28% of labels provided instructions on appropriate utensils to use when feeding the product to the child.

## Discussion

The current study assessed the compliance of PCFs to indicators of appropriate PCF labeling. Compliance was judged against indicators proposed by the Maternal, Infant, and Young Child Nutrition Working Group (MIYCN-WG) of the “10-Year Strategy to Reduce Vitamin and Mineral Deficiencies,” as well as indicators identified from the NBPR, which is based on the international CMBMS on Marketing of Breast milk substitutes in Accra, Ghana (24). The current study observed gaps in compliance across indicators that have relevance for the quality of complementary feeding of children exposed to PCFs.

These finding should, however, be interpreted with consideration that there are no globally accepted standards for the labeling of PCFs. Thus, manufacturer’s labels and the messages/images on them were motivated by marketing and profit-driven decisions. Such decisions may be informed by existing recommendations on child feeding. However, the manufacturers are not required to meet any standards at this time (1, 8). In the absence of appropriate guidance, the MIYCN Working group guidance document presents a useful tool for nation-wide monitoring of PCF labeling. In addition, the current study was carried out in a small geographical area and included only a small number of product labels (*n* = 100). Nevertheless, important gaps related to images on the labels suggest important loopholes that manufacturers can take advantage, albeit subtly, to promote their products in ways, which are inimical to the nutrition of young children.

Given that similar findings regarding PCFs labeling were observed in South Africa (27), there is need for global action to provide guidance on PCFs labeling and marketing. As in the case of South Africa, our findings suggest that even basic labeling expectations regarding readability of the information and language were inappropriately done on the products examined. Leadership by the WHO and UNICEF regarding labeling of PCF is thus warranted to achieve similar consensus that was reached regarding the use of the CMBMS for regulating marketing of formula (17). International leadership to develop appropriate guidelines should be complemented by demonstrated political will, at the country level, to enforce existing regulations.

The development of such global guidance should be informed by and also build upon existing frameworks, such as the case in Ghana where there exists the NBPR. The role of legislation is important since without legal authority, powerful multi-national manufacturers can hold economic sway over developing countries where the enforcement regime is often flawed.

## Conclusion

The labeling practices of PCFs in Ghana are suboptimal and require the Ghana Health Service and other enforcing authorities to enforce the current laws to ensure that labeling of PCFs do not undermine the protection and promotion of optimal infant and young child feeding practices. Labeling practices of particular concern were those on images of children as well as messages that support and protect breastfeeding. The study showed that only 14% of labels assessed showed pictures of young children who were at least 6 months old.

### Recommendations

Labeling practices for PCFs sold in Ghana are inappropriate; manufacturing and vending of PCFs should be regulated to protect and promote optimal breastfeeding. Furthermore, the existing NBPR needs to be revised to include appropriate labeling guidance for PCFs.

The current study has provided evidence, which demonstrates that PCF labeling practices, especially of images on product labels can be misleading, especially in Ghana where significant proportion of caregivers have low literacy levels. Regulatory authorities need to protect the public interest to ensure that the aspects of the law on permissible illustrations on PCF labels are adequately enforced.

Finally, the current study adds to the body of evidence, which requires the WHO and other relevant United Nations agencies to provide leadership and guidance on the inappropriate promotion and labeling of processed foods for infants and young children, as requested by member states in the year 2012 (28).

## Conflict of Interest Statement

The authors declare that the research was conducted in the absence of any commercial or financial relationships that could be construed or misconstrued as a potential conflict of interest have no conflict of interest to declare.
